# Caring for older patients with advanced chronic kidney disease and considering their needs: a qualitative study

**DOI:** 10.1186/s12882-020-01870-1

**Published:** 2020-06-03

**Authors:** Nwamaka D. Eneanya, Allison K. Labbe, Taylor L. Stallings, Shananssa Percy, Jennifer S. Temel, Tamar A. Klaiman, Elyse R. Park

**Affiliations:** 1 Renal-Electrolyte and Hypertension Division, Perelman School of Medicine, University of Pennsylvania, 307 Blockley Hall, 423 Guardian Drive, Philadelphia, PA 19104 USA; 2 Palliative and Advanced Illness Research Center, Perelman School of Medicine, University of Pennsylvania, Philadelphia, PA USA; 3 Department of Psychiatry, Massachusetts General Hospital, Harvard Medical School, Boston, MA USA; 4 Division of Nephrology, Department of Internal Medicine, Massachusetts General Hospital, Harvard Medical School Center, Boston, MA USA; 5 Division of Hematology and Oncology, Department of Internal Medicine, Massachusetts General Hospital, Harvard Medical School, Boston, MA USA

**Keywords:** Advance chronic kidney disease, Advance care planning, Dialysis decision-making

## Abstract

**Background:**

Older patients with advanced chronic kidney disease often do not understand treatment options for renal replacement therapy, conservative kidney management, and advance care planning. It is unclear whether both clinicians and patients have similar perspectives on these treatments and end-of-life care. Thus, the aim of this study was to explore clinician and patient/caregiver perceptions of treatments for end-stage renal disease and advance care planning.

**Methods:**

This was a qualitative interview study of nephrologists (*n* = 8), primary care physicians (*n* = 8), patients (*n* = 10, ≥ 65 years and estimated glomerular filtration rate < 20), and their caregivers (*n* = 5). Interviews were conducted until thematic saturation was reached. Transcripts were transcribed using TranscribeMe. Using Nvivo 12, we identified key themes via narrative analysis.

**Results:**

We identified three key areas in which nephrologists’, primary care physicians’, and patients’ expectations and/or experiences did not align: 1) dialysis discussions; 2) dialysis decision-making; and 3) processes of advance care planning. Nephrologist felt most comfortable specifically managing renal disease whereas primary care physicians felt their primary role was to advocate for patients and lead advance care planning discussions. Patients and caregivers had many concerns about the impact of dialysis on their lives and did not fully understand advance care planning. Clinicians’ perspectives were aligned with each other but not with patient/caregivers.

**Conclusions:**

Our findings highlight the differences in experiences and expectations between clinicians, patients, and their caregivers regarding treatment decisions and advance care planning. Despite clinician agreement on their responsibilities, patients and caregivers were unclear about several aspects of their care. Further research is needed to test feasible models of patient-centered education and communication to ensure that all stakeholders are informed and feel engaged.

## Background

Chronic kidney disease (CKD) among older patients is common in the United States and the prevalence among those aged ≥65 has increased from approximately 3 to 14% in the last two decades [1]. Among patients who ultimately develop end-stage renal disease (ESRD), many are referred by their nephrologists for educational sessions to learn about different treatment options such as dialysis. However, ESRD patient education programs tend to focus on modality type, vascular access, and care setting rather than on how the different treatment modalities may affect patient lifestyles and goals of care [2–4]. Few patients with advanced CKD are aware of treatment options such as conservative kidney management or advance care planning and seldom have an opportunity to discuss preferences with their clinicians or loved ones [5–7]. One recent study of older patients with advanced CKD and ESRD showed that many did not fully understand dialysis and perceived it as the only alternative to death [6].

Emerging models of care demonstrate that integration of palliative care within nephrology practices can help promote conservative kidney management and advance care planning and also improve patient satisfaction among older patients; however, implementation has proved to be challenging [8, 9]. Previous research has demonstrated numerous clinician barriers to providing conservative kidney management and facilitating advance care planning including prognostic uncertainty, inconsistent collaboration between nephrologists and primary care physicians (PCP), and limited knowledge of ESRD treatment options [10–12]. Although there is some literature that explores perceptions of ESRD treatment and end-of-life care options among older patients with advanced CKD and ESRD, it is unclear whether their views are consistent with their clinicians [6, 13–19]. To fill this knowledge gap, we sought to explore the alignment between clinician views of their responsibilities and patient clinical experiences via qualitative interviews with nephrologists, PCPs, patients, and caregivers regarding ESRD treatments and advance care planning.

## Methods

### Study design and participant population

We conducted this qualitative study between 3/2017 and 5/2018 at Massachusetts General Hospital in Boston, Massachusetts. Eligibility criteria included a convenience sample of PCPs and nephrologists who cared for older patients with advanced CKD (aged ≥65 with estimated glomerular filtration rate < 20 as defined by the CKD Epidemiology Collaboration for estimation of GFR) who were not on dialysis [20].

PCPs and nephrologists were recruited via email from primary care and nephrology clinics at Massachusetts General Hospital. All clinicians had a professional working relationship with N.D.E and all clinicians who were approached agreed to participate in the study. Written informed consent was subsequently obtained by the primary investigator (N.D.E, MD – nephrologist, female]). A telephone interview was then scheduled by A.K.L, (PhD – psychologist, female). A study coordinator (S.P, BA - study coordinator, female) approached patients with advanced CKD and their caregivers on the day they were scheduled to receive in-person dialysis education from trained nurses in the outpatient nephrology clinic. None of the patients had a relationship with N.D.E. prior to recruitment. Out of 15 patients who were approached, 3 patients were not interested in the study. Twelve patients were consented, however 2 were withdrawn from the study as one was hospitalized and the other was lost to follow-up. Out of 10 enrolled patients, 5 patients had at least one caregiver who was interested in participating in the study. The study coordinator obtained written informed consent for all participants and scheduled the phone interview. All phone interviews took place in a quiet setting of the participant’s choice. Patient and caregivers were interviewed separately. No other individuals were present during the phone interviews outside of the study participant and interviewer (A.K.L).

Investigative team members, all trained in qualitative methods, (N.D.E, A.K.L and E.R.P [PhD – psychologist, female]) developed both clinician and patient/caregiver semi-structured interview guides (See Additional file 1). Both guides were pilot tested among clinicians (*n* = 3) and patients (*n* = 5) and modified based on feedback prior to use in the study. All interviews were conducted and audiotaped by the same member of the investigative team (A.K.L). There were no field notes recorded during the interview as they were conducted by telephone. One clinician interview (nephrologist) was repeated due to audiotape malfunction. Prior to the interview, none of the participants knew A.K.L or had any knowledge of her research interests. Interviews were conducted among the convenience sample who represented varied perspectives. The Institutional Review Boards at Partners HealthCare and the University of Pennsylvania approved this study.

### Analyses

TranscribeMe transcribed all audio recordings [21]. Transcripts were not returned to participants for comment or correction. Team members N.D.E, A.K.L, and T.A.K (PhD – researcher trained in qualitative methods, female,) initially reviewed all the transcripts and created two codebooks (clinician and patient/caregiver) based on content that arose in the interviews. Transcripts were analyzed using Nvivo (version 12; QSR international, Melbourne, Australia). The codebook was iteratively revised after independent coding of initial transcripts by team members N.D.E, A.K.L, and T.L.S (MS – researcher trained in qualitative methods, female). After consensus meetings with all team members, the final codebooks were used to code the remaining transcripts (clinician transcripts – N.D.E and A.K.L; patient and caregiver transcripts – N.D.E and T.L.S). Emergent themes beyond those included in the interview guide were identified and discussed during the coding process. After reviewing all transcripts, themes were generally consistent across varied perspectives within the sample [22]. We determined that we had reached thematic saturation at this point [14, 22, 23]. Coding discrepancies were resolved via consensus. Participants did not provide feedback on findings.

## Results

**Fig. 1:**
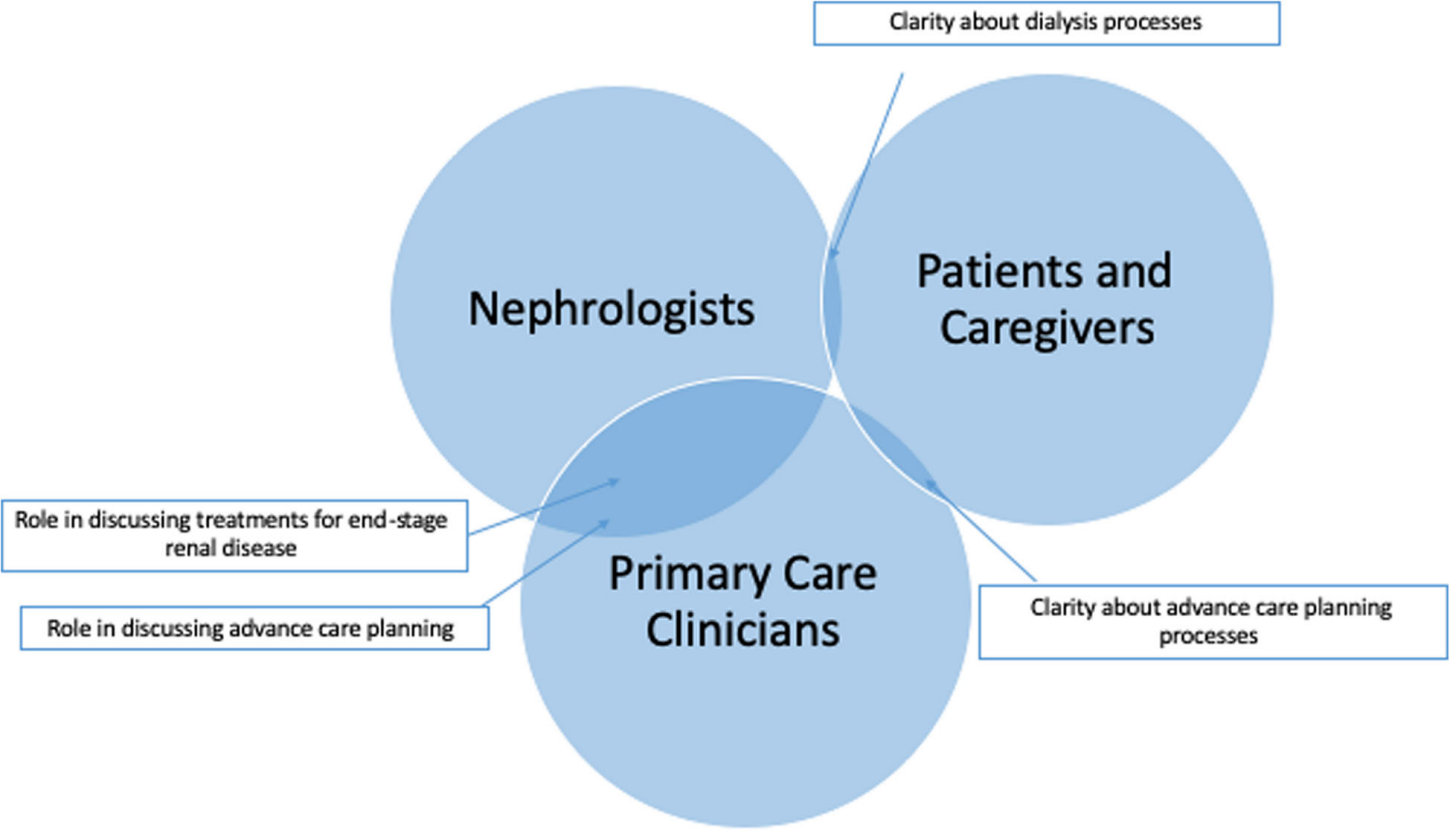
Alignment of clinician and patient/caregiver expectations and experiences

Mean time for clinician interviews (nephrologists *n* = 8, primary care clinicians *n* = 8) was 28 min and 3 s; 12 min and 18 s for patient interviews (*n* = 10); and 11 min and 22 s for caregiver interviews (*n* = 5). Demographic data for all participants are displayed in Table 1. We found a clear difference in alignment of experiences and expectations between clinicians and patients and their caregivers (Fig. 1).

**Table 1:** Participant characteristics

	Clinicians (*n* = 16)	Patients (*n* = 10)	Caregivers (*n* = 5)
Mean age ± SD	44.8 ± 12	73 ± 5.9	62.8 ± 7.7
Female (%)	5 (21)	4 (40)	5 (100)
Mean years of practice ± SD	14.8 ± 11.7	–	–
Race (%)			
White	11 (69)	9 (90)	5 (100)
Black	1 (6)	1 (10)	0
Asian	4 (25)	0	0
Non-Hispanic ethnicity (%)	14 (88)	10 (100)	5 (100)
Highest level of education (%)			
< High school diploma	0	2 (20)	0
High school diploma	0	5 (50)	5 (100)
College degree	0	3 (30)	0
Graduate degree	16 (100)	0	0

### Themes

Three major themes emerged from the data.

#### Dialysis discussions

Nephrologists and primary care clinicians felt that nephrologists’ primary role was to manage all aspects of care specific to kidney disease and initiate dialysis discussions (Table 2). Several nephrologists remarked that they educated all of their patients about dialysis regardless of whether this would be beneficial to the patient. Consideration of lifestyle changes and quality of life were usually not strongly emphasized in dialysis discussions.

**Table 2:** Dialysis discussions

*Nephrologists*	*Primary care physicians*	*Patients and caregivers*
“So my job is to start the process and tell them how to get more information on the time that we’re not together, and then continually follow up with them until they feel good about the decision.” *(Clinician #3)* “The nephrologist should 100% be the-- I think we have the best sense of what that [dialysis] kind of entails. Of course, if there are other physicians and other people, part of the team, I think getting a sense of the patient’s overall goals and functional status and comorbidities is very, very relevant, but this is something that we will ultimately be prescribing and managing. So, I think we should have the central role in it.” *(Clinician #2)* “Yeah... I bring it up in almost all cases. And then if there are glaring issues that I think a patient would suffer more than benefit from dialysis, I point those out because most patients are aware that dialysis is an option. So, I think never bringing it up is not part of my practice because they’re going to wonder about it.” *(Clinician #8)* “I have never had someone who refused to go through dialysis for quality of life purposes.” *(Clinician #1)*	*“*I think the primary care doctor kind of understands the most about the patient’s social situation and I think my role and a primary care doctor’s role is to kind of help patients through the initiation of dialysis, actually, and through the kind of challenges of getting the treatment.” *(Clinician #6)* *“*So, I’ll be thinking about their whole illness, their whole life, how these things would fit in. And I can help them understand kind of what life would be like if they go on dialysis and how I would still be involved in their care.” *(Clinician #12)* “There are, however, other situations where I’ve had this same patient for many, many years and now maybe I referred them to nephrology, but I know the patient better. I know the patient’s family. I know the patient’s trajectory of how this has all developed and I think in those settings, I mean, I think the primary care doctor can always offer knowledge of the patient and the patient’s goals and contribute that to the conversation.” *(Clinician #13)*	*“*The nurse … went over everything with us. They gave my mom a little booklet, which was very good information in it. She explained it would be three times a week. We talked about a fistula, that her getting that, and she did mention that my mom could do it at home, but it would be four times a day and my mother didn’t want to do that.” *(Caregiver #25)* “Only that they’re going to be putting fluid into my belly. It’s an input and output, so you put in the fluid and you take it out, and you put it in.” *(Patient #23)* “I know dialysis is something that you have to-- I think insurance only covers it for three days a week. And so, you have to go every other day, and then you can’t miss it, really. And I guess that’s about it, yeah.” *(Patient #18)* “I’m disappointed that-- I got to do what I got to do. I don’t have a choice.” *(Patient #19)* “I don’t know. I’ve never really thought about it. Well, all I know is that I have kidney failure, and if you had no kidneys, what are you going to do? You’re just going to die. So, the only alternative is dialysis.” *(Patient #31)*

In terms of the role of PCPs, many PCPs and nephrologists commented that PCPs valued providing continuity of care in their patients’ lives – especially in the setting of those who started dialysis and had to establish relationships with new nephrologists. Some PCPs and nephrologists felt the PCP’s primary role was to ensure their patients understood how different treatments would impact their livelihood in addition to advocating for them as they navigated the healthcare system.

With regard to dialysis discussions, patients and their caregivers expressed positive sentiments about the quality of information that was provided and that they also trusted their clinicians’ specific recommendations for dialysis. However, most only commented on being educated about the technical aspects of dialysis. These included modality and dialysis setting, access placement, and frequency of treatments. Many were not aware of alternatives to dialysis treatments and felt hopeless about their lack of options.

#### Dialysis decision-making

Often, nephrologists and PCPs felt that nephrologists were the most knowledgeable about which patients would not do well with dialysis therapy. Many nephrologists used frameworks that included age, and the presence of frailty and/or life-limiting illnesses when considering conservative kidney management (Table 3). Many nephrologists remarked that selecting treatments for renal disease based on these parameters was a central role in their clinical practice. In terms of the role of PCPs, nephrologists deferred to PCPs to integrate treatment decision making with patients’ goals and also provide continuity of care.

**Table 3:** Dialysis decision-making

*Nephrologists*	*Primary care clinicians*	*Patients and caregivers*
“I mean, the typical patients who are not great candidates typically have other illnesses that would-- that are either so life-limiting, or would kind of compromise the feasibility of doing dialysis in them. And probably the one I come up with the most is people with dementia.” *(Clinician #2)* “And so, for example, I would bring it up and if the individual has multiple comorbidities.” *(Clinician #8)* “Again, because they [PCPs] usually have a longer-standing relationship with the patient that they can help basically negotiate what would be the best decision-making at this stage of the patient’s clinical condition and at this stage in the patient’s life.” *(Clinician #7)* “[PCPs] to, I guess be a sounding board. And if the patient has questions or if the patient goes to a primary care doctor and it doesn’t seem like they’re getting what’s going on, I’m hoping that the primary care doctor sort of has their ears open for that and can feed that back to me, so we can get the patient more education.” *(Clinician #3)*	“And then I think yeah, I’m not the ultimate decision maker, so trying to serve as the liaison between the nephrologist and the patient if there’s any disagreement on what might be best going forward.” *(Clinician #10)* “I struggle with this [dialysis decision] because I’m a primary care doctor...Well, I would say if I’m not sure that they’re going to ever need it, if I don’t have the clinical confidence that it’s going to be offered to them. If I don’t think it’s going to be offered, I’m not going to start a conversation about it. So, I guess, usually, the reason that would really keep me from talking about it would be the feeling that the nephrologist is not going recommend it. Yeah.” *(Clinician #12)* “Yeah. You know, in the primary care setting … I think that often these discussions for the average patient are sort of had serially, where the patient goes and talks to a nephrologist, the patient comes talks with the primary care provider. I think that whenever there’s some more complicated decision-making process, for instance dialysis is not clearly of benefit, it is something the patient may opt against reasonably, then that often prompts a more directed discussion with the nephrologist. And the logistics often for folks require some discussion between primary care and nephrology as well.” *(Clinician #11)*	“How long it’s going to be. That’s my concern is, is this going to be once I start it’s going to be forever?” *(Patient #20)* “Well, it affected me. I think of a lot of different things. I feel as though I’m going to lose a lot of my freedom. If I wanted to travel or something, I’m concerned about that. There’s a lot of concerns that I don’t see any real positive outlook on.” *(Patient #18)* “It’s scary. You really don’t know what to expect. Hopefully, it will help her instead of do nothing.” *(Caregiver #25)* “That he’s going to get sicker and issues could happen. I read some different horror stories of different dialysis centers. So, I just hope he gets better and not worse.” *(Caregiver #32)* “Well, I gain that I’m able to live normally, I mean, to the extent where I have to go every other day.” *(Patient #18)* “They told me it would be-- help me out, make me better, hopefully live longer.” *(Patient #21)*

However, although PCPs agreed that they should primarily help patients navigate their renal disease, several PCPs remarked that they struggled with ESRD treatment decisions for their older and frail patients with advanced CKD. For example, some PCPs felt that dialysis may not be the best treatment for their patients, but PCPs also did not feel that this decision was not for them to make. Many commented that deferring this decision to the nephrologist made the most sense. Others felt that decisions about dialysis or conservative kidney management should be made in conjunction with the nephrologist.

Although clinicians agreed about their respective roles in dialysis decision-making with their patients, patients and caregivers did not have a clear understanding of the implications of dialysis and expectations varied. For instance, many expressed concerns about the general effects of dialysis on their lifestyles. Other respondents were not sure what to anticipate from dialysis. Many expressed hopes that dialysis would make them feel better while others longed to live longer and normal lives.

#### Processes of advance care planning

Nephrologists felt that advance care planning discussions should primarily be led by PCPs, and many remarked that they did not feel comfortable leading these discussions (Table 4). Some felt that advance care planning discussions should be conducted in the setting of an interdisciplinary meeting that included the patient, caregivers, and the clinicians most involved in that patient’s care.

**Table 4:** Processes of advance care planning

*Nephrologists*	*Primary care physicians*	*Patients and caregivers*
“I usually kind of put that [advance care planning] in the hands of the primary care physician. I would say I think that’s the cultural norm for a variety of reasons that makes sense. They theoretically are looking at the broader scope of the patient. We tend to be sub-specialists who are consulting. Someone referred the patient to us for a pretty specific reason, mainly kidney failure, or whatever, and so we try to stay within our assigned role of that.” *(Clinician #2)* “I just don’t feel comfortable and I don’t-- I also don’t want to duplicate or maybe contradict if the patient has already discussed this [advance care planning] with a primary care physician or some other providers. They come to see me for nephrology care primarily, so I focus on nephrology with patients. If somebody asks to me to do this, I’d be happy to, but I don’t particularly do it-- actually, I’ve never done it.” *(Clinician #4)* “So yes, I have done some of that. Could I do better? Certainly …. They’re doing that more and more on an inpatient basis. I think the primary care doctors could do a better job because it’s pretty consistent that our patients have not discussed that.” *(Clinician #8)*	*“*I mean, often. I usually do it many times. I mean, I see patients at annuals and follow ups. And usually, at least at their annual, we talk about some of these aspects.” *(Clinician #13)* “Again, like I said before, having a relationship. Again, I think primary care doctors are in the situation where they have hopefully established trusted relationships. I think the nephrologist, as someone who’s sort of seeing somebody for the first time, it’s always hard.” *(Clinician #16)* “The family and the patient and me. And with the backdrop of the nephrologist having given me the information and possibly-- so if you’re asking should the nephrologist be the first person to raise it that is often not the right circumstance …” *(Clinician #15)* “Absolutely. I think anybody who’s going on dialysis, especially late in life or with significant comorbidities, it’s a helpful trigger for doing some advance care planning.” *(Clinician #11)*	“Not really. My husband and I have talked, but no one else has talked.” *(Patient #21)* “We haven’t done one yet, but because she told us that because I’m his wife then I would have a say in it.” *(Caregiver #27)* *“*That was many years ago that I filled that out. I’ve been in and out of the hospital for years. I’m sure that it’s just part of the process.” *(Patient #31)* “I guess if it has to be, it has to be done.” *(Patient #26)* “I think it’s a necessity. People should know and should have the right to go the way they want to go.” *(Caregiver #34)* “When you’re at the point that you know something like that is happening, I guess. If it points that way, I would want to have it before it was too late, I would say.” *(Patient #33)* *“*At the doctor. At the kidney doctor, really. The doctor I see really.” *(Patient #20)* *“*Okay. And so where should this conversation occur? In the kidney doctor’s office …” *(Caregiver #27)*

PCPs agreed with nephrologists about the role of discussing advance care planning and many PCPs felt they should initiate and drive goals of care discussions. Some PCPs also remarked that conducting goals of care conversations in conjunction with nephrologists would be ideal. Some PCPs felt that discussions should occur before or at the same time as renal replacement therapy discussions.

In contrast to clinicians’ clarity about the process of discussing advance care planning, many patients and caregivers had not discussed their end-of-life wishes with their clinicians. Some patients had discussed their preferences with their caregivers and had also completed advance directives. Of those who had completed a health care proxy or living will, many reported they had done so during a hospitalization or before an elective surgery. Regardless of why they completed the form, only a few remarked on the value of having someone speak on their behalf and honoring their wishes if they became incapacitated. Patients and their caregivers also desired goals of care discussions that were honest and included the chances of survival if they were to choose dialysis. Some felt that these discussions should occur earlier in the disease course – before the time dialysis would be needed. Several also described wanting to have discussions with their family members at their nephrologists’ office or in their homes.

## Discussion

Among nephrologists, primary care clinicians, older patients with advanced CKD and their caregivers, perspectives and expectations about ESRD treatments and advance care planning varied. Furthermore, although clinicians were mostly aligned on their respective roles with regard to communication and care delivery, patients and caregivers were unclear about several aspects of dialysis, alternatives to treatment, and advance care planning. Lastly, patients and caregivers specifically desired more information about treatment choices and advance care planning from their nephrologists.

In the United States, the infrastructure to effectively deliver nephrology care to older patients with advanced chronic kidney disease continues to evolve [24]. Conservative kidney management, which incorporates non-dialytic therapy that aims to slow progression of disease and treat a wide range of symptoms, is an approach that many clinicians are still learning about [5, 25, 26]. We found that both nephrologists and PCPs agreed that nephrologists should take the initiative in determining which patients are eligible for dialysis. However, PCPs struggled with dialysis decisions that had been made for patients who they personally felt would not fare well. Our results are consistent with Parvez et al. who found that, compared to nephrologists, PCPs were less knowledgeable about conservative kidney management treatment effectiveness and less confident about selection of suitable candidates [10]. However, this study also showed that nephrologists had difficulty with determining which of their patients would benefit from conservative kidney management. Ladin et al., described the “interpretative” approach to conservative kidney management (e.g., nephrologists serve as a treatment decision navigator for patients) as the best example of shared decision-making given that patients’ values and goals are incorporated into the process [27]. However, in this same study, a minority of nephrologists actually presented conservative kidney management as an option to their patients. As we found that PCPs felt strongly about guiding their patients through the decision-making process, a collaborative model in which both the PCP and nephrologist assume distinct roles (e.g., information provider and treatment decision navigator, respectively) could potentially improve ESRD decision-making for this patient population [12, 28–30].

Integrative models of primary and specialty care have also been described to improve the rates of goals of care discussions and advance care planning among seriously ill patients [31, 32]. In our study, nephrologists and PCPs felt that although advance care planning was an important aspect of care for their older patients, most believed that PCPs should lead these discussions. These findings counter work from O’Hare et al. where they found that among physicians from different specialties (e.g. nephrology, cardiology, primary care, etc.), there was an unclear understanding of who should carry out advance care planning [11]. We also found that the few patients and caregivers who had goals of care discussions or had completed advanced directives, had done so in an inpatient setting. However, a recent study showed that standard initiation of advance care planning in primary care practices was effectively implemented across a large integrated health care system using staff training and electronic medical record enhancements [33]. Notably, most clinicians in our study described primarily communicating about their shared patients through the electronic medical record or email. Therefore, structured primary care interventions that utilize telecommunication as an adjunct to in-person clinic visits could promote interdisciplinary collaboration and circumvent systemic barriers to advance care planning such as lack of time and fragmentation of care [11, 34–37].

Perhaps the most striking finding from our study was the lack of clarity about ESRD treatment options and subsequent effects on livelihood as well as advance care planning among patients and their caregivers. The majority of patients and their caregivers seemed confident only in their knowledge of the *process* of dialysis treatments and advance directive completion. In particular, older patients with advanced CKD have been shown to have knowledge gaps with regard to prognostic awareness and disease progression [17]. However, our findings showed that patients were also in need of more information about the direct effects of ESRD treatments on their quality of life and longevity. Patients and caregivers also desired more information from their nephrologists regarding advance care planning – especially in the setting of dialysis decisions. In one recent study of older adults with advanced CKD, patients described poor communication with their nephrologists about their disease as a source of worry about their future health and misconceptions about their illness [17]. Another study showed that skewed perceptions about ESRD and end-of-life treatments could lead to more intense care at the end of life for patients with kidney disease [14]. These findings, in addition to our own, highlight the need for improved communication between nephrologists, PCPs, and their patients to fulfill the psychosocial needs of patients in addition to technical information requests. Current clinician training programs on how to effectively conduct goals of care discussions with seriously ill patients with kidney disease show self-reported improvement and confidence in communication skills [38]. However, more research is needed to also evaluate the quality of communication and whether concerns regarding illness and treatment effects are adequately addressed among older patients and their caregivers.

This study has a few limitations. First, the modest sample size limits transferability of our findings to clinicians, patients, and caregivers in different settings. Second, although we reached thematic saturation, it is possible that we could have identified additional observations with more interviews. Third, conversations about ESRD treatments with patients and caregivers mostly centered around dialysis options and we were thus unable to glean insights into perspectives about non-dialytic treatments. Lastly, as we only included nephrologists and PCPs, we were unable to make inferences about experiences and perceptions among other members of the health care team.

## Conclusions

This is the first study to our knowledge to comprehensively examine experiences around ESRD treatments and advance care planning discussions between clinicians, patients with advanced CKD and their caregivers. Our findings identify an important tension between clinicians’ perceptions of communication and care delivery and unmet needs of patients and their caregivers. Further research is needed to develop and implement collaborative models of care to holistically care for older patients with advanced CKD and improve understanding of conservative kidney management and advance care planning.

## Supplementary information

**Additional file 1.**

## Data Availability

The datasets used and/or analyzed during the current study are available from the corresponding author on reasonable request.

## Abbreviations

CKD: Chronic kidney disease
ESRD: End-stage renal disease
PCP: Primary care physician
