# Supplementary material for: Caring for older patients with advanced chronic kidney disease and considering their needs: a qualitative study
Source: BMC Nephrol. 2020 Jun 3;21:213. doi: 10.1186/s12882-020-01870-1 (PMC7271389; doi:10.1186/s12882-020-01870-1)
Supplement: Supplementary file 1 — Additional file 1. [file 12882_2020_1870_MOESM1_ESM.docx]

**Clinician Interview Guide:**

*Introduction*

Hello Dr. _______. My name is ________ and I am working on a study of advance care planning for patients with advanced chronic kidney disease.

Thank you for your willingness to participate in this important study.

This 20 minute interview is to explore your experience and perceptions of caring for patients with advanced chronic kidney disease. .

Your answers will be audio-recorded, pooled with responses from other clinicians in this study, and de-identified in our analyses.

As a thank you, you will receive a $10 gift certificate for your participation

If we use any of the comments you share you will not be personally identified.

Is this ok? □ Yes (If Yes, turn on recorder)

Let’s get started...please interrupt me if you have questions.

There are a variety of choices of therapies for patients with advanced chronic kidney disease. I would like to ask about the process you go through when deciding between conservative management versus dialysis.

Tell me about the process that you go through when considering dialysis verses conservative management.

a. What patient characteristics trigger discussions about dialysis initiation?

b. When do you consider having discussions about initiation of dialysis?

c. What do you say to them?

d What patient characteristics make you NOT have these discussions about dialysis initiation?

2. Think about some patients who were not good candidates for dialysis and discussions about treatment for their kidney failure. Tell me about these discussions.

a. Did you bring up these discussions or were they patient-initiated?

b. What do you say to them?

c. What made this conversation easier?

d. What made this conversation more difficult?

3. What do you think your role is in having dialysis decision-making discussions with the patient?

a. How do you go about this role? What approach/style do you use? What are your responsibilities?

b. Who else do you think should initiate and reinforce these discussions?

4. What has been your experience with communicating your thoughts about your patient’s advanced chronic kidney disease prognosis with their primary care provider/nephrologist? (If they say they have not had any experiences, skip questions a and b)

a. When do you have this conversation with this provider?

b. What do you say to them?

c. What do you expect is their role?

d. What happens when you disagree about your patient’s prognosis with their primary care provider/nephrologist?

Let’s talk about another patient issue. Sometimes when thinking of life-sustaining treatments like dialysis, some may also start thinking about advance care planning.

5. Have you initiated the subject of advance care planning with your patients with advanced chronic kidney disease?

If yes:

Please tell me about the process you go through in initiating these discussions.

a. What patient characteristics trigger advance care planning discussions?

b. When do you consider having discussions about advance care planning?

c. What do you say to them?

d What patient characteristics make you NOT have these discussions about advance care planning?

e. What makes these conversations easier?

f. What makes these conversations more difficult?

If no:

a. Why not?

6. Have you specifically discussed treatment options such as CPR or mechanical ventilation?

If yes:

a. Do you bring up these discussions or were they patient-initiated?

b. What do you say to them?

If no:

1. Why not?

7. I would like your suggestions about the best way to talk about advance care planning for care for your patients with advanced kidney disease?

a. When do you think this should be done?

b. Where do you think this should be done?

c. Who should be a part of this discussion?

d. What are your thoughts about follow-up discussions about this topic?

8. Do you have any other thoughts that you would like to share about these conversations?

Thank you for your time and for sharing your experiences. We greatly appreciate your efforts to help improve education for patients with advanced kidney disease.

**Patient Interview Guide:**

*Introduction*

Hello, may I speak with ________________________?

This is ________________, following up on the research study that your kidney doctor told you about.

We are starting a study that includes people who have been referred to learn about dialysis. Your experiences as a person who has been referred for dialysis education are valuable to us; it would be great if you could answer some questions that will help us educate patients better in our research study.

This will take 20-30 minutes.

You will receive $20 as a way of saying thank you.

Is now an OK time to talk about this?

If NO, make appointment for call-back: ____________________

If YES, Great! I am going to ask you some questions. There are no right or wrong answers. Just give me your opinions.

If you don’t want to answer a question, just tell me.

So that I can be sure to get down all of your comments***,*** I will audio record our conversation. Nothing on the recording will identify you personally and the recording will only be shared with study staff.

If we use any of the comments you share you will not be personally identified.

Is this ok? □ Yes If Yes, turn on recorder

Let’s get started...please interrupt me if you have questions.

1. I understand that you have recently been referred for dialysis education and may need dialysis.

a. What were you told about the recommendation about dialysis?

b. Do you think you need it? Why or why not?

c. Who made this recommendation?

(i).What other clinicians recommended dialysis , if any?

(ii). Do you feel that you trust your clinician(s) that made the recommendation?

d. What was that like for you to hear this recommendation?

2. What do you know about dialysis?

a. What are your expectations about dialysis?

b. What do you hope to gain with dialysis treatment?

c. What are your concerns about dialysis?

d. How does it make you feel to think about dialysis?

Thank you for sharing your experiences with dialysis conversations. Sometimes, when people talk about dialysis treatments, they also talk about care they would desire if they were to become very sick after starting dialysis. I would like to know more about your experiences with these types of conversations.

3. Have any clinicians talked to you about planning for care at the end of life if you were to become very sick?

If yes:

a. Who brought up this conversation?

b. What did they say?

c. What was that like for you to talk about this?

If no, skip to question 4

4. Have any clinicians talked to you about filling out a health care proxy form, or a form that specifies who you would want to make your medical decisions if you could not make them on your own?

If yes:

a. What was said in the conversation?

b What do you think about this form?

c. Are you thinking of filling one out? Why or why not?

(i). (if patient has already completed a health care proxy form): Why did you choose to complete this form?

If no, skip to question 5

5. Have any clinicians talked to you about filling out a living will or MOLST (Medical Orders for Life-Sustaining Treatment) form or a form that specifies what you want in terms of life-sustaining therapies (e.g. CPR, mechanical ventilation, etc)?

If yes:

a. What was said in the conversation?

b. What do you think about filling out these forms?

c. Are you thinking of filling one out? Why or why not?

(i). (if patient has already completed one of these forms): Why did you choose to complete this form?

If no, skip to question 6

6. If you could design the ideal conversation for needing dialysis, how would it go?

a. What setting should this conversation occur?

b. What things should be discussed?

c. Who should be part of this conversation?

d. When should this conversation occur?

7. If you could design the ideal conversation for planning ahead for care at the end of life if you were to become very sick, how would it go?

a. What setting should this conversation occur?

b. What things should be discussed?

c. Who should be part of this conversation?

d. When should this conversation occur?

8. Is there anything else that you would like to share today?

Thank you so much for you time and sharing your experiences with me. This information will be helpful to help educate people who have been diagnosed with severe kidney disease. I encourage you to talk to your kidney doctor and primary care physician if you have questions about your health.

**Family Member/Friend Interview Guide:**

*Introduction*

Hello, may I speak with ________________________?

This is ________________, following up on the research study that your family member/friend’s kidney doctor told you about.

We are starting a study that includes family members and friends of people who have been referred to learn about dialysis. Your experiences as a person who knows someone who has been referred for dialysis education are valuable to us; it would be great if you could answer some questions that will help us educate patients better in our research study.

This will take 20-30 minutes.

You will receive $20 as a way of saying thank you.

Is now an OK time to talk about this?

If NO, make appointment for call-back: ____________________

If YES, Great! I am going to ask you some questions. There are no right or wrong answers. Just give me your opinions.

If you don’t want to answer a question, just tell me.

So that I can be sure to get down all of your comments***,*** I will audio record our conversation. Nothing on the recording will identify you personally and the recording will only be shared with study staff.

If we use any of the comments you share you will not be personally identified.

Is this ok? □ Yes If Yes, turn on recorder

Let’s get started...please interrupt me if you have questions.

1. I understand that your family member/friend have recently been referred for dialysis education and may need dialysis.

a. What were you told about the recommendation about dialysis?

b. Do you think they need it? Why or why not?

c. Who made this recommendation?

(i). What other clinicians recommended dialysis , if any?

(ii). Do you feel that you trust their clinician(s) that made the recommendation?

d. What was that like for you to hear this recommendation?

2. What do you know about dialysis?

a. What are your expectations about dialysis?

b. What do you hope your family member/friend gains with dialysis treatment?

c. What are your concerns about dialysis?

d. How does it make you feel to think about dialysis?

Thank you for sharing your experiences with dialysis conversations. Sometimes, when people talk about dialysis treatments, they also talk about care they would desire if they were to become very sick after starting dialysis. I would like to know more about your experiences with these types of conversations.

3. Have any clinicians talked to your family member/friend about planning for care at the end of life if they were to become very sick?

If yes:

a. Who brought up this conversation?

b. What did they say?

c. What was that like for you to talk about this?

If no, skip to question 4

4. Have any clinicians talked to your family member/friend about filling out a health care proxy form, or a form that specifies who they would want to make their medical decisions if they could not make them on your own?

If yes:

a. What was said in the conversation?

b What do you think about this form?

c. Is your family member/friend thinking of filling one out? Why or why not?

(i). (if the patient has already completed a health care proxy form): Why did your family member/friend choose to complete this form?

If no, skip to question 5

5. Have any clinicians talked to your family member/friend about filling out a living will or MOLST (Medical Orders for Life-Sustaining Treatment) form or a form that specifies what your family member/friend want in terms of life-sustaining therapies (e.g. CPR, mechanical ventilation, etc)?

If yes:

a. What was said in the conversation?

b. What do you think about filling out these forms?

c. Is your family member/friend thinking of filling one out? Why or why not?

(i). (if patient has already completed one of these forms): Why did your family member/friend choose to complete this form?

If no, skip to question 6

6. If you could design the ideal conversation for needing dialysis, how would it go?

a. What setting should this conversation occur?

b. What things should be discussed?

c. Who should be part of this conversation?

d. When should this conversation occur?

7. If you could design the ideal conversation for planning ahead for care at the end of life if your family member/friend were to become very sick, how would it go?

a. What setting should this conversation occur?

b. What things should be discussed?

c. Who should be part of this conversation?

d. When should this conversation occur?

8. Is there anything else that you would like to share today?

Thank you so much for you time and sharing your experiences with me. This information will be helpful to help educate people who have been diagnosed with severe kidney disease. I encourage you to talk to your family member/friend’s kidney doctor and primary care physician if you have questions about their health.
